# In Memoriam; Prof. Wm. Proctor, Jr

**Published:** 1874-04

**Authors:** 


### IN MEMORIAM.

It is with feelings of deepest regret—both personal and professional regret—that we announce to our readers the sudden death of our friend and former teacher, William Procter, Jr., Professor of Practical Pharmacy in the Philadelphia College of Pharmacy.

"Professor William Procter, Jr., died February 10th, of heart disease, at the age of 57. On the preceding evening he had lectured at the college, and retired near midnight, apparently in his usual health; about half an hour later he breathed his last."

For a period of thirty-seven years Prof. Procter has been quietly and unobtrusively, but earnestly and most devotedly, laboring for the building up and advancement of pharmaceutical science and art. Although but little known to the medical profession at large, perhaps no man in the country has done so much of faithful and valuable service in the decenniel revision and perfecting of the National Pharmcopœia as he; and though his name does not appear upon the title-page of that great encyclopædia of pharmacology, the United States Dispensatory, the rich and enduring results of his life's labors are to be found upon almost every page of the ponderous volume. For many long years he was the intimate friend and adviser upon the one hand, and the untiring, faithful servant upon the other, of the venerable Dr. Geo. B. Wood, its illustrious co-author and compiler.

Procter was an humble, modest and retiring laborer in science, day by day adding his valuable services in tilling the field, content and satisfied to do the work, and see others bedeck themselves with the flowers and enjoy the fruits. At a banquet given in honor of the American Pharmaceutical Association in Boston in 1859, the health of Prof. Procter was offered in a bumper of champagne, and accompanied by a brief, but just, tribute to his eminent labors and personal worth. Never have we seen a man so overcome; the crimson blush upon the cheek of a sensitive and bashful miss of fourteen could not have evidenced a more sensitive shrinking from the public gaze. For many long years he occupied the tripod in the *American Journal of Pharmacy*, and carried that periodical to a most enviable position among the scientific exponents of this country.

Peace to his ashes! and may a grateful posterity do that honor to his distinguished services to science, and to his personal worth, which he so timidly shrank from in life.

